# Self‐healing encapsulation and controlled release of vaccine antigens from PLGA microparticles delivered by microneedle patches

**DOI:** 10.1002/btm2.10103

**Published:** 2018-10-30

**Authors:** J. Maxwell Mazzara, Lukasz J. Ochyl, Justin K. Y. Hong, James J. Moon, Mark R. Prausnitz, Steven P. Schwendeman

**Affiliations:** ^1^ Department of Pharmaceutical Sciences University of Michigan Ann Arbor MI; ^2^ Biointerfaces Institute, University of Michigan Ann Arbor MI; ^3^ Department of Biomedical Engineering University of Michigan Ann Arbor MI; ^4^ School of Chemical and Biomolecular Engineering Georgia Institute of Technology Atlanta GA

**Keywords:** controlled release, microneedles, PLGA, vaccine delivery

## Abstract

There is an urgent need to reduce reliance on hypodermic injections for many vaccines to increase vaccination safety and coverage. Alternative approaches include controlled release formulations, which reduce dosing frequencies, and utilizing alternative delivery devices such as microneedle patches (MNPs). This work explores development of controlled release microparticles made of poly (lactic‐*co*‐glycolic acid) (PLGA) that stably encapsulate various antigens though aqueous active self‐healing encapsulation (ASE). These microparticles are incorporated into rapid‐dissolving MNPs for intradermal vaccination.

PLGA microparticles containing Alhydrogel are loaded with antigens separate from microparticle fabrication using ASE. This avoids antigen expsoure to many stressors. The microparticles demonstrate bi‐phasic release, with initial burst of soluble antigen, followed by delayed release of Alhydrogel‐complexed antigen over approximately 2 months in vitro. For delivery, the microparticles are incorporated into MNPs designed with pedestals to extend functional microneedle length. These microneedles readily penetrate skin and rapidly dissolve to deposit microparticles intradermally. Microparticles remain in the tissue for extended residence, with MNP‐induced micropores resealing readily. In animal models, these patches generate robust immune responses that are comparable to conventional administration techniques. This lays the framework for a versatile vaccine delivery system that could be self‐applied with important logistical advantages over hypodermic injections.

## 1 INTRODUCTION

While vaccines represent our strongest weapons against contagious disease, several obstacles still limit their maximum potential. For example, most vaccines require booster doses to induce protective levels of immunity. Besides, the large molecular size of vaccine antigens often prevents oral administration, and hypodermic injections are necessary to elicit the desired immune response. The reliance on repeated hypodermic injections creates many logistical challenges, such as difficulties with storage, disposal, and administration via healthcare professional. This not only increases costs, but also decreases availability, particularly in developing nations. If the full booster schedule is not administered, an individual may not develop protective immunity. Furthermore, hypodermic needles are designed for intramuscular (i.m.) delivery. However, the muscle has a low level of resident antigen‐presenting cells (APCs), thus requiring higher doses than would be needed when compared to more APC‐dense tissue such as the skin.^1^, ^2^, ^3^, ^4^

In an effort to increase the availability of vaccines and improve worldwide vaccination coverage, the next generation of vaccines should reduce reliance on hypodermic injections. This could be achieved through multiple approaches. One option is developing single‐administration vaccines, which may offer protective immunity from a single dose.^5^ A second concept, which could be accomplished separately or in tandem, is to utilize alternative delivery devices such as microneedle patches (MNPs) that avoid the logistical hurdles of hypodermic needles and are also more patient‐friendly.^4^, ^6^, ^7^, ^8^, ^9^

A promising route to developing safe single‐administration vaccines is through controlled antigen release.^5^, ^10^, ^11^ This can be pulsatile to mimic current prime‐boost paradigms,^12^, ^13^ or continuous to mimic a naturally developing infection.^14^, ^15^ In either case, the goal is to develop protective immunity via extended/delayed antigen exposure from a single administration. A common approach for controlled release is to encapsulate the active ingredient in a bio‐erodible polymer such as a poly(lactic‐*co*‐glycolic acid) (PLGA).^16^, ^17^, ^18^ While this approach has generated commercial success with various small molecules and peptides, it has not historically translated well to biomacromolecules such as protein antigens.^19^ This is primarily due to the harsh stresses experienced during fabrication and sterilization of, and release from, the microparticles, which are known to damage sensitive proteins.^20^, ^21^, ^22^, ^23^, ^24^

**Figure 1:**
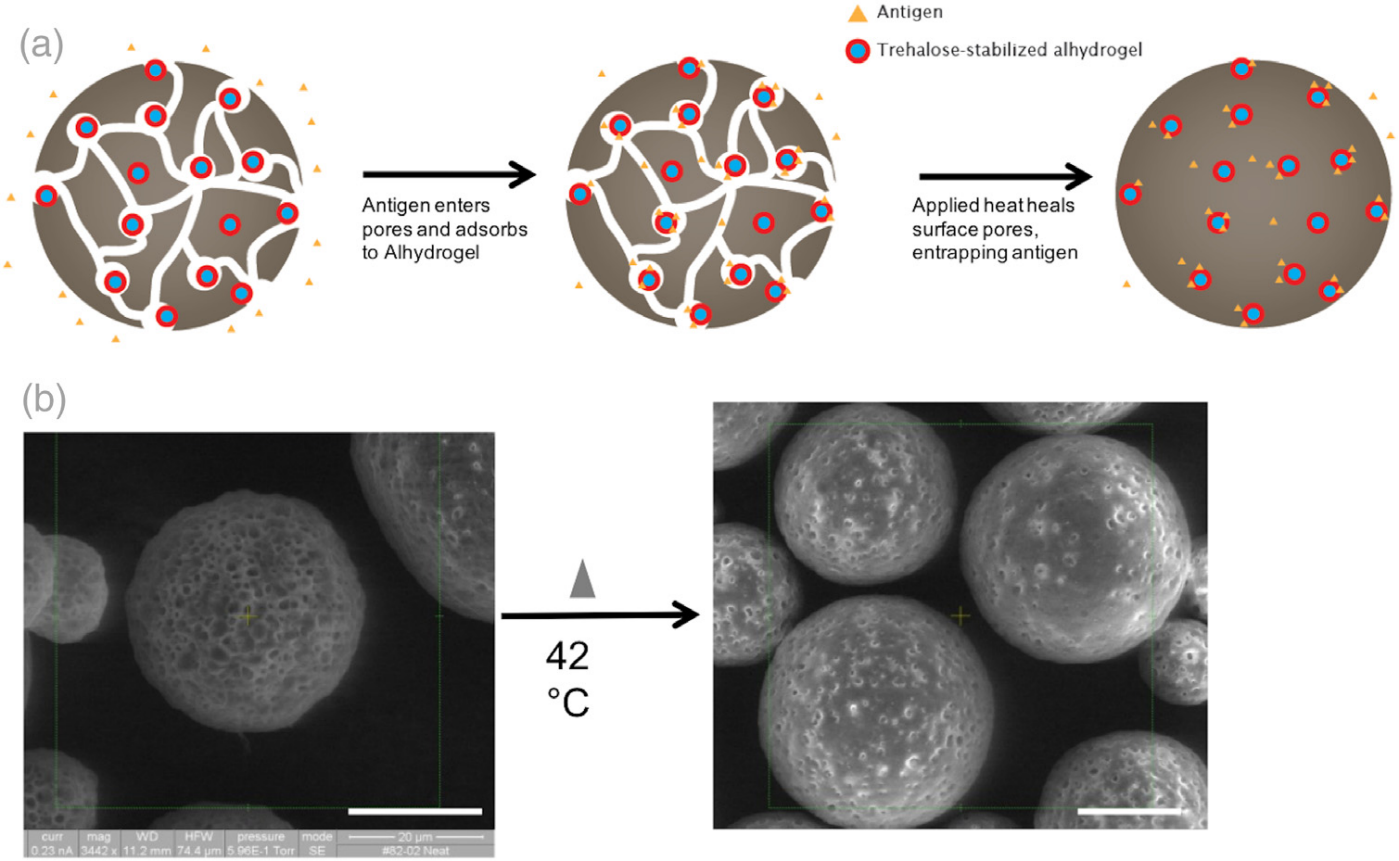
(a) Schematic of aqueous ASE loading method. Porous microparticles containing trehalose‐stabilized Alhydrogel are fabricated and freeze‐dried. Microparticles are soaked in an antigen solution, antigen enters the pores and adsorbs to Alhydrogel. The solution is then mildly heated, healing the pores and entrapping the antigen. Microparticles can then be collected, washed, and utilized. (b) *SEM* images of porous ASE microparticles after fabrication and lyophilization (left), and after loading and partial self‐healing (right). Scale = 20 μm

Newer approaches however allow for the separation of microparticle fabrication from the act of protein loading, thus allowing stable protein to be encapsulated.^24^, ^25^, ^26^ This method, termed active self‐healing encapsulation (ASE), employs a protein‐trapping agent inside the microparticles, which draws protein into the microparticles from an aqueous solution at high efficiency, followed by a dynamic self‐healing process of microparticles' surface pores that trap protein inside the microparticles (Figure 1a).^24^, ^26^, ^27^ This method is well suited for protein antigens, and has great potential for the development of a single‐administration controlled release vaccine delivery system.

Whereas these PLGA controlled release systems have potential for reducing overall dosing requirements, they still rely on hypodermic needles for administration, which are often disliked by patients, require serious storage and disposal considerations, and generally must be administered by a healthcare professional.^1^, ^6^, ^8^, ^28^ MNPs are an attractive alternative, as they do not suffer from many of the obstacles mentioned above. In brief, MNPs are typically patches containing small sharp projections (∼100–1,000 μm) that penetrate into superficial layers of the skin and deliver a therapeutic payload intradermally (i.d.).^4^, ^6^, ^7^, ^28^, ^29^ Due to their small size, the patches cause little or no pain and generally no bleeding.^30^, ^31^ They also have reduced storage/disposal requirements, and may dissolve entirely after application, leaving behind no biohazardous sharps waste, which reduces risk of accidental stick or reuse.^32^ Furthermore, MNPs are generally preferred by patients over traditional hypodermic injections, and can be successfully self‐administered without a healthcare professional.^8^ Lastly, by delivering the payload to the skin, they take advantage of the potent intradermal immune system, which can generate stronger responses than what is typical of the muscle, or can generate equivalent responses from lower doses.^1^, ^4^, ^33^, ^34^

Explored here is the combination of controlled protein antigen release from PLGA microparticles loaded via ASE with the logistical and immunological benefits of administration via microneedles. PLGA microparticles are first fabricated without antigen present, containing only the common vaccine adjuvant Alhydrogel, and trehalose as a stabilizing and pore‐forming excipient. A variety of different vaccine antigens are then loaded into the same microparticle formulation using the ASE loading paradigm. These microparticles are then incorporated in a MNP, where the controlled antigen release behavior is evaluated in vitro. These patches readily penetrate skin and then rapidly dissolve to deliver the microparticles i.d. where they reside to release antigen. This system has great potential as a self‐applied and versatile controlled release vaccine delivery system.

## 2 RESULTS AND DISCUSSION

### 2.1 Fabrication and evaluation of ASE‐loaded PLGA microparticles

The formulation parameters of the ASE PLGA microparticles were selected to produce spherical, porous microparticles within the desired size range (10–60 μm) that demonstrated self‐healing when incubated in solution above the hydrated PLGA glass‐transition temperature (*T*_g_).^27^, ^35^ The *T*_g_ of the dry microparticles was 46.5 °C, while after hydration this value dropped to 32.6 °C (Supporting Information). The observed *T*_g_ depression of the hydrated microparticles is expected because of the well‐known plasticization effect of water on polymers.^36^ The microparticles were well formed and highly porous as observed via scanning electron microscopy (*SEM*; Figure 1b). The hydrated microparticles had a volume‐weighted mean diameter of 35.0 μm; larger than the limit up to which phagocytic cells can internalize a particle.^37^ Thus, encapsulated antigen will likely be hidden from the immune system until it is released from the microparticles as soluble or adjuvant‐bound protein.

The major advantage of the ASE loading strategy is it allows formulation optimization of the preformed microspheres in the absence of protein. This reduces the amount of potentially expensive protein wasted during pilot formulation studies. Any microparticles larger than the desired size could be excluded from the final product with particle sieves without wasting antigen.

To evaluate the microparticles' ability to load different antigens, dry and unloaded microparticles were co‐incubated with various antigen solutions during a loading gamut that included 48 hr at 42 °C. After this period, the pores on the microparticle surface had partially or fully healed (Figure 1b). This serves to close off some diffusion pathways for soluble antigen, and slows the inital burst release. While many different loading conditions, including varying antigen concentration, volume, or maximum temperature, successfully produced antigen‐loaded microparticles, it was found using ovalbumin (OVA) as the model antigen that 0.5 ml of a 1 mg/ml OVA solution incubated with 20 mg of microparticles for 2 days at 4 °C, followed by 1 day at room temperature, and 2 days at 42 °C produced the best combination of wt/wt antigen loading and encapsulation efficiency (EE%) for this formulation (Supporting Information). Also, a variety of antigens, both model and clinically relevant, were successfully encapsulated into the exact same formulation of microparticles—that is, alterations to the microparticles were not needed to accommodate different antigens. The changes in wt/wt loading generally correlated with the antigens' affinity for the Alhydrogel that was included in the formulation (Table 1). It should be noted that the time used for self‐healing encapsulation in the above protocol is undesirably long. Means to accelerate the loading via the use of plasticizers in the polymer, for example, are currently under investigation.

**Table 1:** Multiple antigens can be loaded into the same microparticle formulation using the ASE technique

Antigen	Microparticle loading % (*wAv*)	Adsorption capacity to Alhydrogei (mg/mg)
OVA	1.64 ± 0.03	1.25 ± 0.02
rHBsAg	2.25 ± 0.11	2.66 ± 0.29
rPA^a^	0.90 ± 0.02	1.21 ± 0.05
Fl‐V^a^	0.83 ± 0.14	0.81 ± 0.06
TT^b^	1.38 ± 0.20	N/A

a Twenty percent trehalose added to the loading solution to improve temperature stability.

b Data from Ref. [24], which utilized similar microparticle formulations and an identical loading approach. ±*SEM*.

Alhydrogel is a common vaccine adjuvant currently included in many different vaccines.^38^, ^39^ It was loaded into the microparticles at 3.5% (theoretical wt/wt). The adjuvant binds to antigens to create colloidal particles, thus extending their residence time and increasing phagocytosis.^38^, ^39^, ^40^ Here, Alhydrogel also acts as an agent to preferentially sequester antigen inside the microparticles. That is, during incubation antigen diffuses into the microparticle pores where it binds Alhydrogel to become trapped inside the microparticles (i.e., loading) before the surface pores heal under elevated temperature. Because Alhydrogel can bind to most proteins at pH above the protein's pI, it offers a versatile system to work with many different antigens without the need to change the microparticle formulation. The primary additional consideration is for thermoliable antigens—in this case the anthrax antigen rPA and the plague antigen F1‐V. In order for these antigens to remain stable during the loading conditions, an appropriate stabilizer, such as 20% wt/wt trehalose added to the antigen solution was necessary, as has been previously reported.^41^ While the addition of trehalose as an excipient stabilized the antigen, it also interferes with loading. When 20% trehalose was added to OVA controls, loading was reduced by 45% (*data not shown*). Thus, future studies focused on formulations with OVA and Hepatitis B surface antigen (rHBsAg).

### 2.2 Development of microneedle patches containing ASE microparticles

**Figure 2:**
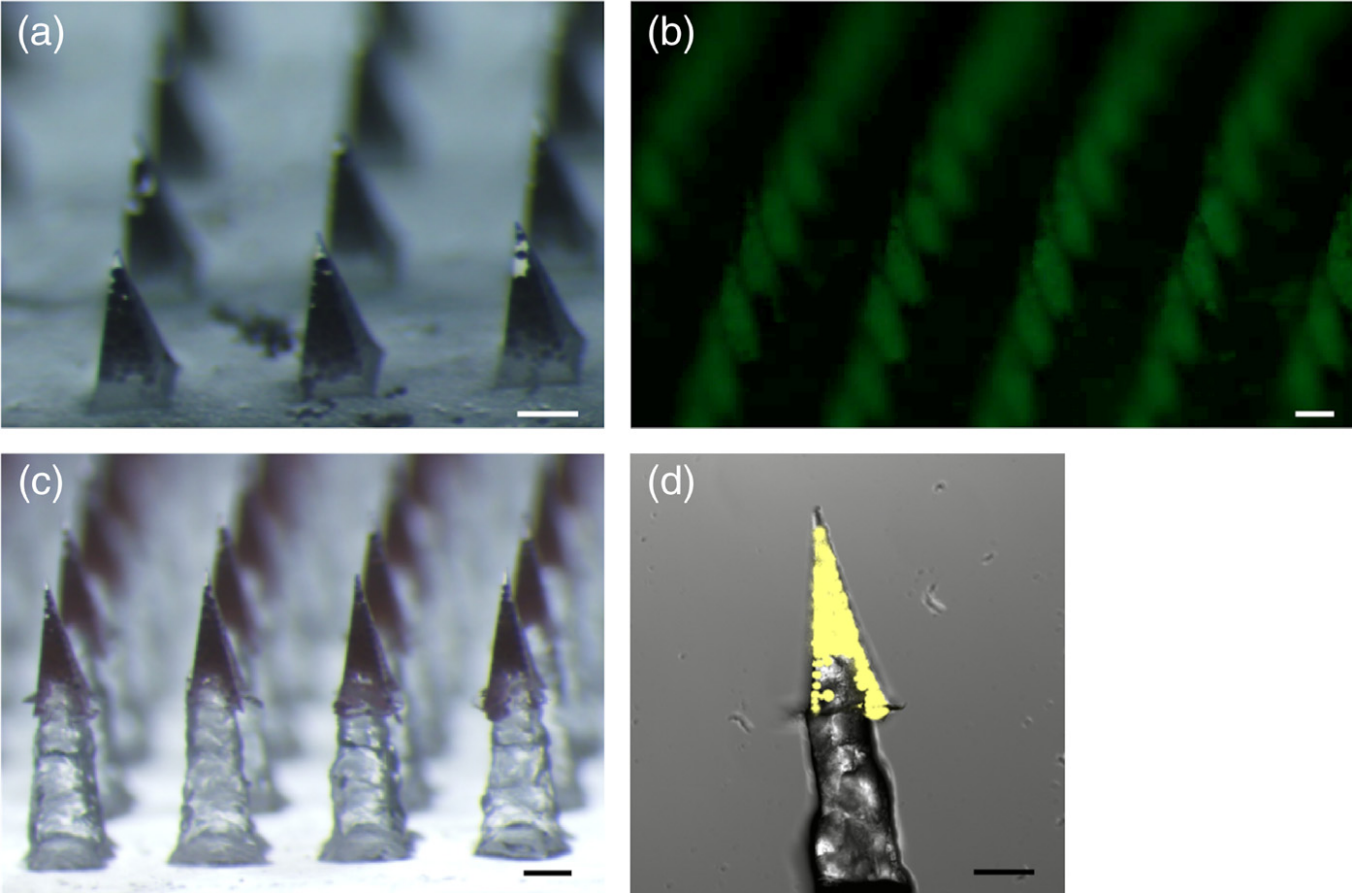
Micrographs of microparticle‐loaded microneedle patches. (a) Standard patch, (b) fluorescent micrograph of standard patch loaded with fOVA‐loaded microparticles, (c) pedestal patch with sulforhodamine B added to the first PVA/sucrose cast, and (d) confocal image of individual pedestal microneedle containing microparticles loaded with fOVA. Scale = 250 μm

Microparticles were then incorporated into microneedles composed of highly water‐soluble materials (i.e., polyvinyl alcohol [PVA] and sucrose). In this way, the microneedles dissolve quickly in the skin (thereby allowing the MNP to be removed from the skin within a few minutes), leaving the microparticles deposited as a depot within the skin. MNPs were prepared using “standard” microneedles (Figure 2a) and using “pedestal” microneedles that were mounted atop a pedastal to improve microneedle insertion into deformable skin (Figure 2c). The PVA/sucrose composition was selected as it maximized solid content of the filling solution, while also providing an acceptable viscosity.

Previous work has explored incorporating nanoparticles into microneedles,^42^, ^43^, ^44^ but incorporation of microparticles into microneedles has received limited attention.^29^ In this study, the microparticles are large and thus remain extracellular during release. Furthermore, this is the first time microparticles loaded via the ASE loading technique have been utilized in a MNP, which is expected to improve antigen stability.^24^

When making standard patches, microparticles could be readily observed in the microneedles, with few particles in the backing (Figure 2a,b). The process was also easily adapted to include a pedestal design that increased the functional length of the microneedles while keeping the microparticles localized to the microneedle portion (Figure 2c,d).

The standard and pedestal MN patches contained approximately 244 and 208 μg of microparticles, respectively (Table 2). The difference was likely due to the extra manipulation required of the pedestal patches. Using the model antigen OVA, which loads into the microparticles at 1.6% (wt/wt), this corresponded to a final antigen dose of 4.0 and 3.4 μg/patch for standard and pedestal patches, respectively (Table 2). Also, each patch is expected to contain less than 10 μg of alhydrogel—well below the FDA limit of 0.85 mg/dose, even if multiple patches were administered. The antigen loading would be expected to change when using different antigens. To adjust dosage, several options are possible, such as changing the number of microneedles in the array, using multiple patches, or diluting the microparticles with a packing excipient. It may be challenging to incorporate additional microparticles in this size range into a microneedle without changing the overall geometry.

**Table 2:** Microparticle and antigen mass contained within a single standard or pedestal microneedle patch. % MPs delivered represents the percent of microparticles delivered to the tissue after a 20‐min application on live mice. ±*SEM*

Patch design	Microparticle mass/patch	OVA mass/patch (μg)	% MPs delivered
**Standard**	244 μg *±* 8	4.0 ± 0.0	25% ***±*** 11
**Pedestal**	208 **μg** *±* 9	3.4 ± 0.0	55% ± 8

Pedestal‐based microneedles are helpful for overcoming the elasticity of the skin and ensuring more full penetration/insertion of the microneedles into the tissue. Using a standard pyramidal/conical microneedle design, it is common for only 25% of the total microneedle volume to be dissolved or deposited in the tissue.^45^, ^46^, ^47^ The pedestal design utilized here was crafted using three‐dimensional (3D)‐printed master parts that were re‐cast using soluble materials. While 3D printing lacks the micron‐scale precision and accuracy of photolithograpy, presice dimensions and smooth surfaces are not generally required of the pedestal part, so 3D printing was an effective means of reducing fabrication costs and time. In addition, by creating a pedestal patch that is fully soluble, it eliminates considerations for disposal of biohazardous waste versus other two‐part systems.^47^, ^48^ While the standard microneedles had a height of 600 μm, and the pedestal part was 800 μm tall, the final tip‐to‐base height of the pedestal patches was 1,183 ± 6 μm, suggesting roughly 200 μm of overlap between the pedestal and the microneedle, as confirmed by confocal imaging (Figure 2d).

### 2.3 In vitro controlled release

**Figure 3:**
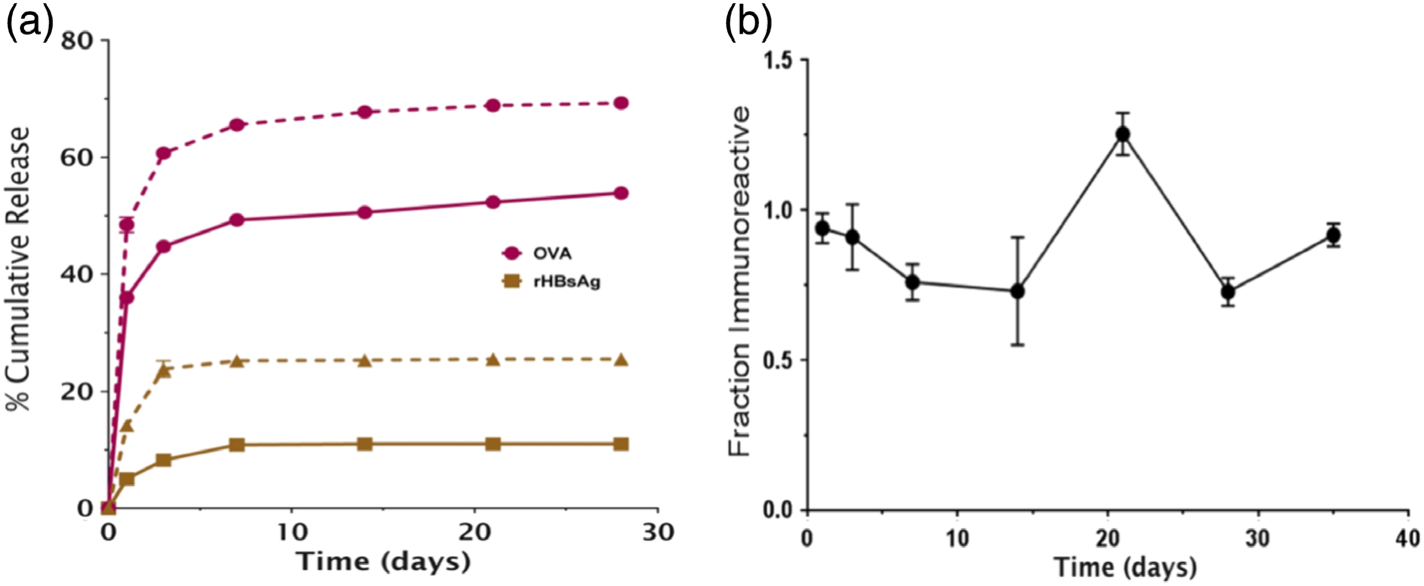
Microneedle patches encapsulating microparticles demonstrate controlled release of stable soluble antigen over approximately 1 month in vitro. (a) Controlled release of soluble OVA and rHBsAg from MNPs (solid) and microparticles (for control, dashed). (b) Immunoreactivity of OVA after release from MN patches, defined as ratio of concentration as determined by ELISA to concentration determined via SEC. *n* = 3, ±*SEM*

In vitro release was evaluated for both independent microparticles and MNPs containing microparticles using both model (OVA) and clinically relevant (rHBsAg) antigens. For MNPs, encapsulated microparticles were first liberated from the PVA/sucrose microneedle matrix by dissolving and rinsing with cold dI‐H_2_O to avoid interference with the antigen signal. Soluble antigen release from MNPs was observed to occur over 2–4 weeks. This included an initial burst release followed by a slight linear phase. After this period, no additional soluble antigen was detectable. During this phase, ∼60% of encapsulated OVA, and ∼10% of rHBsAg were released (Figure 3a). The difference between the two antigens' release profiles is likely due to differences in their predominant binding mechanism to the Alhydrogel inside the microparticles. Antigens can bind Alhydrogel through two dominant mechanisms; reversibly through electrostatic interactions, and irreversibly through ligand exchange.^49^, ^50^ OVA binds primarily through electrostatic interactions,^49^ thus a larger percentage is expected to desorb from the Alhydrogel and diffuse out of the microparticles during this phase. rHBsAg, however, binds primarily through ligand exchange.^51^ Thus, a lower percentage desorbs and more remains inside the microparticles as a particulate complexed to Alhydrogel.^49^, ^52^ It is noteworthy that in vivo, the dissolution and clearance of the PVA/sucrose binding material would be anticipated to take additional time. Thus, the early stage of antigen release is expected to occur slower in vivo than under the in vitro test described above.

The stability of the antigen released during this early phase was evaluated by comparing antigen concentration as determined by size‐exclusion chromatography (SEC) to that determined via an enzyme‐linked immunosorbent assay (ELISA) method (Figure 3b). Stability, particularly at the early timepoints, was near 100%, with little or no decreases at later timepoints. This suggests the combination of the ASE technique along with the presence of stabilizing excipients in the microparticles and MNPs successfully stabilized the antigen during the loading, microneedle fabrication, and freeze‐drying phases. Release from microparticles not incorporated into MNPs was generally similar, but with a slightly larger burst release and higher total percent soluble release (Supporting Information).

To confirm that the remaining fraction of antigen (antigen that did not desorb from Alhydrogel and release from the microparticles as soluble antigen) was still inside the microparticles and had not released as a soluble aggregate or degradation product, microparticles were subjected to total nitrogen analysis after 35 days of in vitro release (Table 3). After day 35, the standalone microparticles had released ∼70% of encapsulated OVA. As the polymer and other excipients are nitrogen‐free, the total nitrogen content can be correlated back to protein content. Roughly 27% of encapsulated protein was recovered (97% total recovery). This strongly suggests the fraction of antigen that is not released during the soluble release phase is remaining inside the microparticles as a ligand‐bound particulate complexed with Alhydrogel, although protein aggregation could not be ruled out.

**Table 3:** The fraction of antigen not released from microparticles in vitro during the soluble release phase can be accounted for via nitrogen analysis. Approximately 70% of encapsulated OVA was released as soluble antigen by day 35 from micropaticles. The remaining samples mass was found to contain approximately 27% of total encapsulated OVA, for 97% total recovery. ±*SEM*

% Soluble OVA release (day 35)	% Remaining (N2 analysis)	Total OVA recovery 1
69.9% ± 0.0	26.9% ± 0.0	96.8% ± 3.5

**Figure 4:**
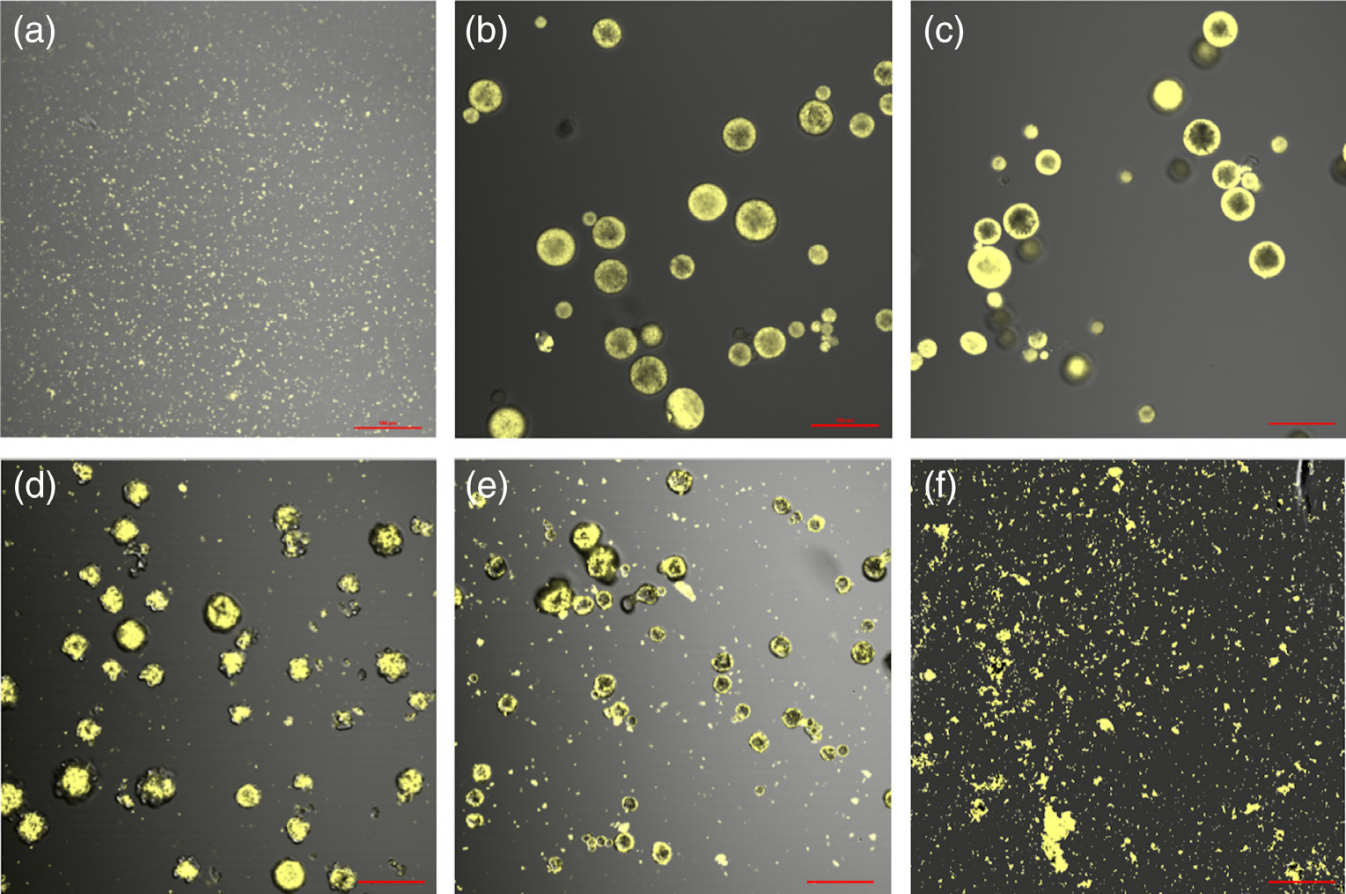
Laser fluorescent confocal images of the microparticles and released Alhydrogel‐fOVA complex during release of Alhydrogel‐fOVA complex from ASE microparticles in vitro. (a) Alhydrogel‐fOVA complex without encapsulation. fOVA‐loaded ASE microparticles after, (b) 7 days, (c) 14 days, (d) 21 days, (e) 28 days, and (f) 42 days of in vitro release at 37 °C. Scale = 100 μm

To evaluate the release characteristics of this remaining antigen fraction, a fluorescently‐labeled OVA (fOVA) was encapsulated into microparticles using the ASE technique. Figure 4a shows the colloidal particles formed by adsorbing fOVA onto Alhydrogel—small particulates no more than a few microns in diameter. Figure 4b,f show microparticles loaded with fOVA after 1, 2, 3, 4, and 6 weeks of in vitro release (after washing away any soluble antigen released). After one and 2 weeks, the microparticles showed no obvious signs of bulk degradation, and the florescent signal was still localized to the microparticles only. After 3 weeks, however, degradation of the polymer microparticles was apparent both in confocal and *SEM* images (Supporting Information). As this happened, mass loss of the polymer occurred and larger pores began to form. This allowed the Alhydrogel‐fOVA complex to escape, which was visible outside the microparticles. This was more apparent at week 4, where the complex was now more visible, and heavy microparticle degradation was obvious. By week 6, the microparticles were fully degraded and the remaining fraction of Alhydrogel‐complexed antigen was released and available for presentation to the immune system. Again, because of the larger size of these microparticles, antigen still encapsulated inside the microparticles is hidden from the immune system until release.

### 2.4 Skin penetration and microparticle delivery

**Figure 5:**
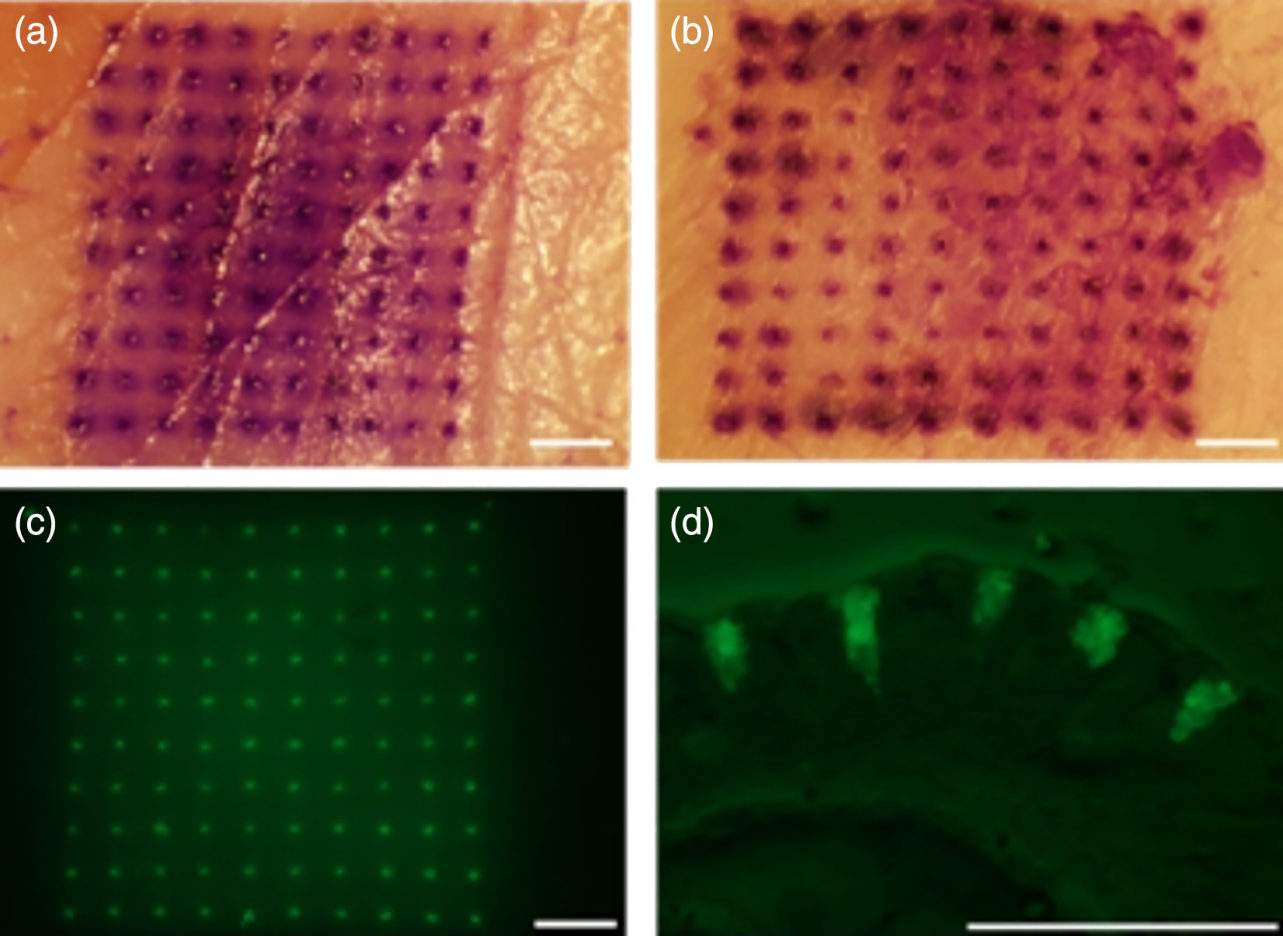
Microneedle patches efficiently penetrate the skin and deposit microparticles intradermally. (Top) Micrographs of excised porcine skin after application and staining of penetration sites. (a) Standard patch, (b) pedestal patch. (bottom) fluorescent micrographs of tissue after application of pedestal patch containing fOVA‐loaded microparticles. (c) Overhead, (d) cross‐sectional. Scale = 1 mm

To evaluate skin penetration, excised porcine inner ear tissue was used. Standard and pedestal patches were pressed into taut skin with the thumb. Standard patches produced a full 100 clearly identifiable microchannels, while pedestal patches produced an average of 98 ± 2 (*n* = 5, ±*SEM*) microchannels (Figure 5a,b). This suggests the patches possess the mechanical integrity necessary to penetrate skin tissue.

To verify that after the microneedles penetrate skin they dissolve i.d. to deliver microparticles, pedestal MNPs were fabricated with microparticles loaded with fOVA. The resulting MNPs were applied as above, but the patches were allowed to remain in the tissue for 20 min to dissolve. After removing the patches, the tissue was fluorescently imaged to visualize the microparticles (Figure 5c). The fluorescence was localized to the 10 × 10 grid pattern, strongly suggesting the microneedles dissolve i.d. and release the microparticle payload, and the microparticles do not spread out either on the surface of the skin or within the tissue. Afterwards, the tissue was frozen and cryosectioned to visualize cross‐sections of the skin at the application site. Figure 5d shows a representative cross‐section of the tissue, and confirms that microparticles had been i.d. deposited via the MNPs. Together, Figures 5c,d suggest that microparticles are not left on the surface of the skin where they would be inactive, but rather are deposited below the stratum corneum, mostly in the dermis.

After removing the partially dissolved patches from the tissue, it was apparent that some microparticles had not been deposited and remained on the patch after administration (Supporting Infomation). The fraction left in the patch could not be determined gravimetrically, as the patches picked up a considerable amount of tissue and hair. Rather, a GPC method was developed to quantify the mass of polymer left on the patch after administration. As shown in Table 2, the standard patches only delivered 25% of the microparticles (consistent with previous results, when presented),^45^, ^46^ while the addition of the pedestal improved this significantly, to 55%. In addition to the elasticity of the skin, insertion is likely limited by rapid dissolution of the microneedle tip, which could quickly become dull after insertion and prevent further tissue penetration. To further improve delivery, slower dissolving and materials could be investigated, possibly coupled with more advanced microneedle‐fabrication methods.

### 2.5 Skin resealing and in vivo microparticle tracking

A potential concern for advancing MNP technologies is the submillimeter pores introduced in the skin by application of the patch. If these micropores do not close quickly the potential for infection may exist, although prior reserarch suggests this risk is small.^53^ While several studies have investigated the kinetics of skin resealing, the existing literature focuses on solid, nondissolving‐type MNPs that do not deposit any material in or otherwise occlude the micropores.^54^, ^55^, ^56^ Thus, it was necessary to explore the skin resealing kinetics after application of the MNPs used here to evaluate if the microparticles, or the PVA/sucrose microneedle matrix, affected the skin's ability to close the micropores. To evaluate skin resealing, trans‐epithelial water loss (TEWL) was measured, as it correlates well to the barrier properties of the skin.^57^

Four styles of patches were evaluated: (a) a non‐dissolving MNP of equivalent geometry that was made of high *Mw* poly(lactic acid) (PLA) (fabricated as previously reported^58^) and did not deposit material in the skin, (b) the microparticle‐loaded dissolving pedestal MNPs explored above, (c) dissolving pedestal MNPs that contained nanoparticles rather than microparticles (median diameter = 7.1 μm),^59^ and (d) a dissolving pedestal MNP that did not contain any microparticles (vehicle).

**Figure 6:**
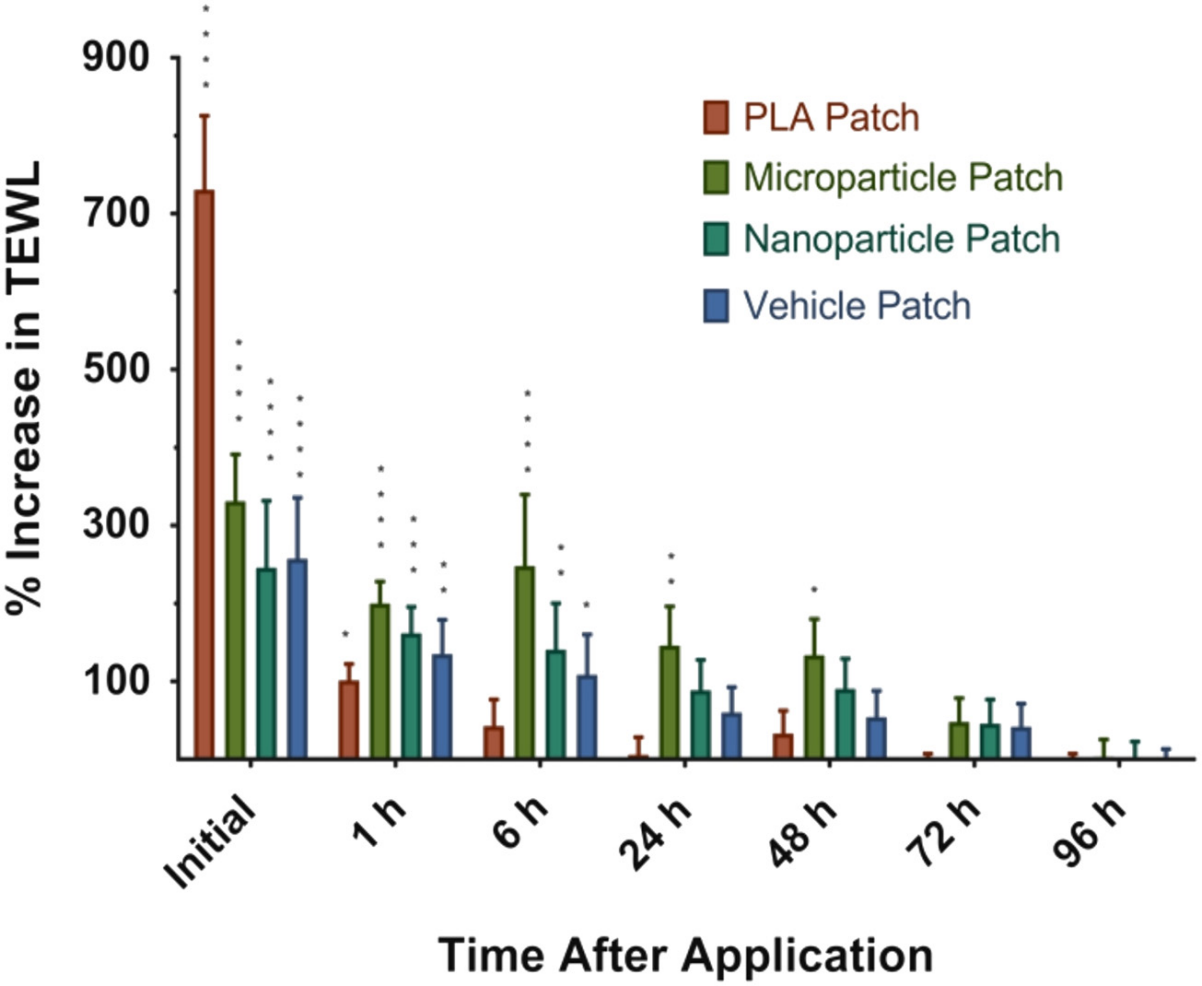
Murine skin resealing as measured by TEWL after in vivo application of various microneedle patches. Unoccluded micropores made by nondissolving PLA patches reseal rapidly, while micropores with deposited material reseal slower, with larger occlusions taking the longest. All values are compaired against a needle‐free application control using Fisher's LSD test. ****p < .0001, ***p < .001, **p < .01, *p < .05. ±*SEM*

Immediately after application, TEWL values for all test groups rose significantly (Figure 6). The PLA patches generated a higher response than the other groups, likely because the stronger, nondissolving material allowed for the creation of a larger/deeper wound not filled with dissolved material or particles.

Within 6 hours of application, micropores introduced by the PLA patches had largely resealed. This is consistent with previous literature suggesting pores made from solid nondissolving MNPs reseal quickly.^54^ Micropores from vehicle patches and from patches containing smaller nanoparticles mostly resealed by the end of the first day, whereas those from the microparticle patches resealed between the second and third day. These data suggest that material deposited in the skin by dissolving microneedles acts as an occlusion and slows the skin resealing process, and that resealing is further slowed by larger particles as compared to smaller ones or only soluble material. However, the skin still resealed within a resonable timefrime, likely encapsulating the microparticles in the dermal space. Additional studies are needed to determine the relationship between skin resealing and possible infection or leakage of microparticles out of the skin.

While penetration and microparticle deposition studies are useful to determine how well MNPs deposit their payload when applied, it is also important to determine the behavior of the microparticles and antigen in the skin over time. While it is generally understood that soluble materials are readily delivered to the circulation and/or lymphatics, the behavior of larger biodegradable depots is less well characterized. To evaluate this, microparticles were again loaded with fOVA and fabricated into pedestal MNPs. Patches were applied to shaved mice which were imaged over time to evaluate the strength and localization of the fluorescent signal. Values were compared against i.d. injected microparticles and soluble OVA.

**Figure 7:**
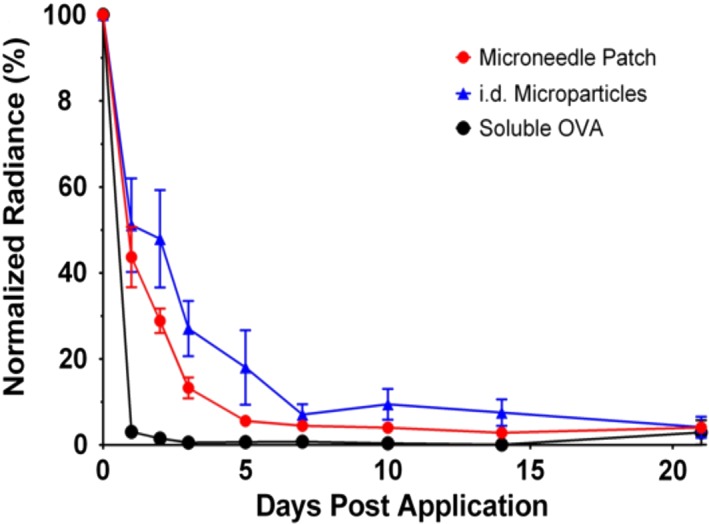
fOVA‐loaded ASE microparticles remain in the skin for several days following intradermal administration from microneedles or Mantoux injection (i.d.), with i.d. soluble OVA as control, as determined by normalized radiance quantification of fOVA signal at the application site. *n* = 8, ±*SEM*

After administration, the application site was highly visible through fluorescent imaging, with individual micropores identifiable (Figure 7). Over the next 3–10 days, the application site retained its fluorescence for patches and injected microparticles. After only 1 day, however, the soluble antigen signal was heavily attenuated and was lost entirely by day 3. As this retention is longer than the time required to reseal the skin, it suggests that microparticles deposited by the MNPs are not quickly pushed out of the skin either by the rapid turnover of the epidermal layer,^60^ or by the general movement of the animals. The signal from MNP‐deposited microparticles was slightly attenuated compared with injected microparticles, possibly due to some loss from the surface or from the animals cleaning the application site, which may have removed additonal microparticles.

### 2.6 Immunizations via ASE microparticles and microneedles

To determine if the microparticles delivered hypodermically or via a MNP stimulate an immune response, mice were dosed with microparticles containing OVA or rHBsAg by multiple routes of administration. Two MNPs were applied to the shaved dorsal flank, while an equivalent delivered dose of antigen‐loaded microparticles were hypodermically injected either i.d. or i.m. Control groups consisted of equivalent delivered antigen doses of Alhydrogel‐adsorbed antigen (positive control), soluble antigen, or MNP containing antigen‐free microparticles (negative control/sham). Booster doses were given 21 days after the priming immunization.

**Figure 8:**
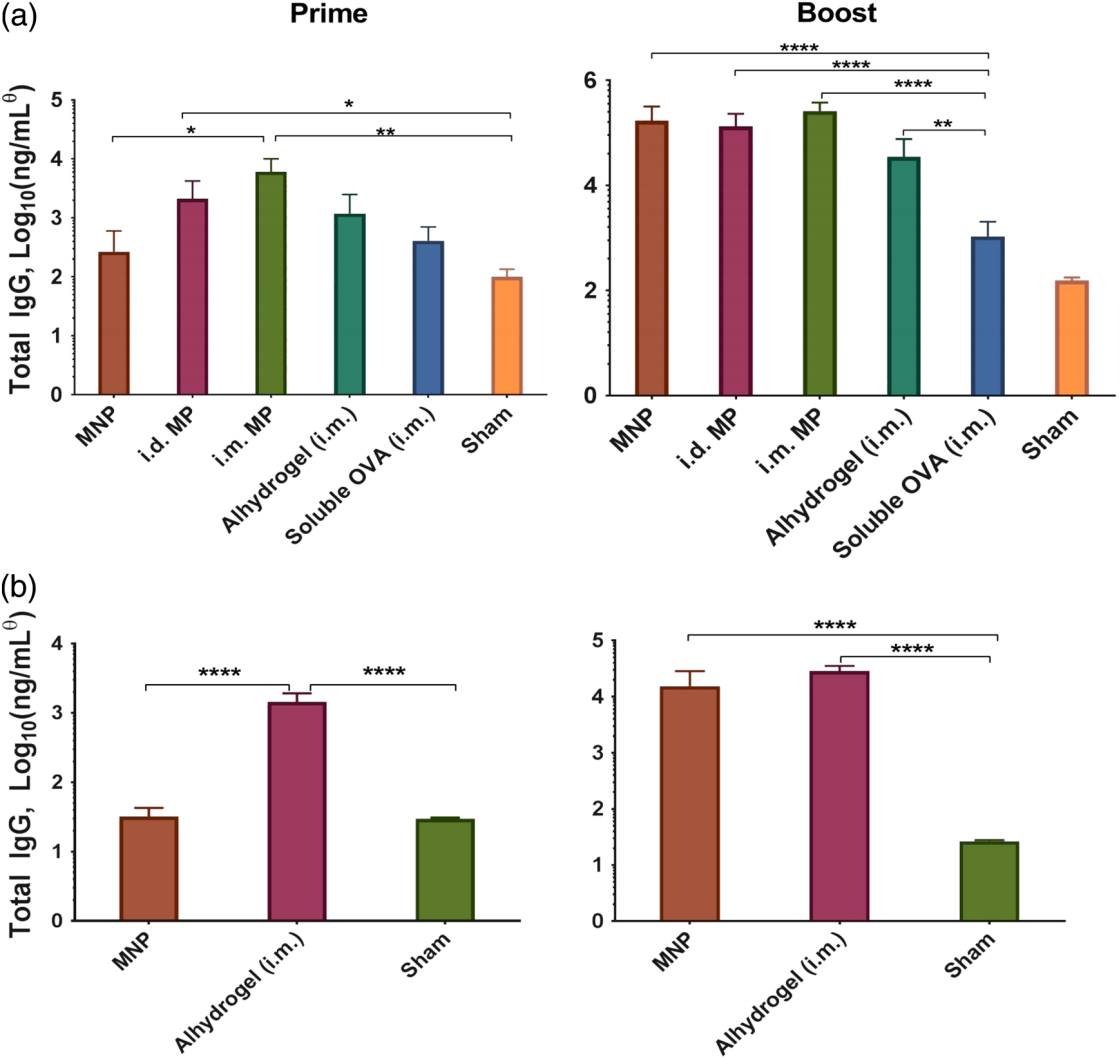
ASE microparticles and MNPs generate robust antibody responses. Serum IgG levels at day 20 (left, prime) and day 42 (right, boost). (a) OVA‐immunized groups, (b) rHBsAg‐immunized groups. (c) Concentrations were determined using an IgG1 standard, and may not be absolute for other IgG isotypes. ****p < .0001, ***p < .001, **p < .01, *p < .05. *n* = 5, ±*SEM*

On day 42, blood was drawn and analyzed for anti‐OVA or anti‐rHBsAg total IgG serum levels, as well as IgG1 and IgG2c, which are indicators of Th2 and Th1‐type immunity, respectively.^61^ For both antigens, all microparticle and/or MNP‐dosed groups showed high antigen‐specific total IgG levels compared with the sham and soluble OVA groups, and were as‐good‐as or better than the conventional vaccine group with Alhydrogel‐adsorbed antigen (Figure 8a,b). Similar trends were observed for IgG1, whereas only i.d. and i.m. injected OVA‐loaded microparticles elicited weakly significantly elevated IgG2c levels (Supporting Information). Given that Alhydrogel‐adsorbed antigens do not frequently generate Th1 responses, and coupled with high levels of IL‐10 levels produced by restimulated splenocytes (Supporting Information), these data suggest that delivery using microparticles and MNPs are capable of generating robust Th2‐type immune responses that perform as‐well‐as or better than conventional vaccination approaches in mice. Furthermore, this approach was readily translatable to different antigens without any changes to the formulation or fabrication process, as evidenced by high IgG levels for both OVA and rHBsAg.

Blood was also drawn and analyzed on day 20, 1 day before booster doses). In this case, microparticles, but not MNPs, showed significantly higher antigen‐specific IgG levels compared to controls. The delay of onset for the MNPs is likely the result of a combination of factors including: (a) additional time required to dissolve and clear the PVA/sucrose binding material, (b) hydration of the freeze‐dried microparticles, and (c) a slight delay in controlled release kinetics between the microparticles and MNPs (Supporting Information). This difference was even more apparent in the rHBsAg groups. At day 20, no response was observed in the groups dosed with MN patches, while the Alhydrogel‐adsorbed group already showed a response. As was shown in Figure 3, the rHBsAg released more slowly (less released in the soluble phase), and thus the response takes longer to develop. However, research suggests that slower releasing antigens generate stronger final immune responses than quick releasing antigen.^15^

These results support further scientific development of hypodermic needle‐free vaccination via ASE microparticle‐containing MNPs, and that the ASE microparticles used in the MNPs may be a useful method for sustained exposure of antigen to the immune system. The MNPs produced responses that were generally equivalent to i.d. injection of microparticles, but did not rely on a hypodermic needle for injection. This seemingly minor detail actually has enormous consequences for improving vaccination coverage for reasons mentioned above, including higher patient acceptability, self‐application, and easier storage/disposal. It is unexpected that i.m. injection of microparticles produced equivalent or occasionally stronger responses than i.d./MN administration, as this trend is typically reversed in the existing literature.^33^, ^42^

The controlled release potential of these polymer‐based delivery systems was also apparent. For example, rHBsAg was shown to release more slowly than OVA from the microneedles in vitro (Figure 3). Before booster doses, it appeared that Alhydrogel‐adsorbed rHBsAg was producing a more robust IgG response, whereas after boost the responses were nearly equivalent. The faster releasing OVA, on the other hand, generated a response more quickly and surpassed the conventional formulation by day 42. This trend may suggest that the slower release of antigen could delay the development of the immune response, but may also lead to the production of a stronger response once release is complete. This is also true of the comparison between the MNPs and i.d. microparticles, as the patches were shown to release antigen more slowly than free microparticles.

## 3 CONCLUSIONS

The vaccine delivery system developed here combines three major components to form a new platform that has multiple advantages over traditional vaccine delivery. First, it utilizes PLGA microparticles loaded via the ASE‐loading paradigm. This system maintains antigen stability by avoiding antigen exposure to the protein‐damaging stresses present during traditional microparticle fabrication/antigen encapsulation. Also, by utilizing the common vaccine adjuvant, Alhydrogel, as the ASE agent that draws antigen into the microparticles, it adds versatility to the system. That is, many antigens that bind to Alhydrogel and are or can be made reasonably temperature stable can be loaded into the same microparticle batch without necessitating any changes to the formulation. Second, the system relies on controlled antigen release via PLGA microparticles, which have the potential to perform as‐well‐as if not better than traditional prime‐boost vaccine schedules. Removing the need for multiple injections is one of the largest obstacles to improving worldwide vaccination coverage, and thus is an important component of the system developed here. Lastly, by incorporating the antigen‐loaded microparticles into a soluble MNP, multiple logistical, and scientific advantages become apparent. These patches are smaller, can be self‐administered, and will dissolve completely to avoid creating biohazardous sharps waste. They are also more likely to be preferred by patients, as they generate minimal or negligible pain and bleeding and are unlikely to induce needle‐phobia. MNPs also utilize the powerful intradermal immune system, which may be more advantageous than traditional i.m. delivery. While additional modifications to the system could further improve its utility, this work lays a foundation for a self‐administered vaccine system that is applicable to a variety of vaccines and thus disease states.

## 4 MATERIALS AND METHODS

### 4.1 PLGA microparticle fabrication and loading

Antigen‐free porous PLGA microparticles were prepared via a water‐in‐oil‐in‐water (w/o/w) solvent evaporation method adapted from methods previously reported.^24^ About 350 mg PLGA 50:50 (i.v. = 0.60 dl/g, ester terminated) was dissolved in 1 ml dichloromethane. The inner‐water phase was prepared by concentrating Alhydrogel (2%, Invivogen) to 6.35% via centrifugation and removal of excess solution, then 8% wt/vol trehalose was added and the slurry was mixed. 0.2 ml of the inner‐water phase was added to 1 ml of the dissolved polymer phase, then homogenized for 1 min at 17 k rpm on a Tempest I.Q.^2^ Sentry Microprocessor to create the primary w/o emulsion. The theoretical alhydrogel loading was thus 3.5% (wt/wt). Two milliliter of a 5% (wt/vol) PVA solution was then added to the primary emulsion and vortexed for 50 s. Lastly, the resulting w/o/w emulsion was poured into 100 ml of a 0.5% (wt/vol) PVA solution and hardened under rapid stirring for 3 hr. The resulting microparticles were collected and passed through between 60‐ and 10‐μm sieves in series and washed with ddH_2_O to remove excess PVA. Excess liquid was removed and the microparticles were freeze‐dried for 48 hr.

Microparticles were loaded with antigens using ASE.^24^, ^26^ Flocculated antigens (OVA and rPA) were resuspended at a stock concentration of 1 mg/ml in 10 mM MOPS buffer, pH 7.4. F1‐V and rHBsAg were first buffer exchanged from PBS to MOPS, then brought to a 1 mg/ml stock in MOPS. A total of 0.5 ml of 1 mg/ml antigen solution was added to 20 mg microparticles, protected from light and rotated for 2 days at 4 °C, 1 day at room temperature, and 2 days at 42 °C. After incubation the suspension was centrifuged and the supernatant was removed and saved for analysis via HPLC/UPLC‐SEC and/or ELISA. Microparticles were either then used directly to create MNPs, or freeze‐dried for independent use. Loading and encapsulation efficiencies (EE%) were determined using the following formulas using the lost mass of antigen from the loading solution compared against positive controls.

$$\%\frac{\mathrm{wt}}{\mathrm{wt}}\text{loading}:\frac{\text{mass of antigen encapsulated by microparticles}}{\text{mass of microparticles}}\times 100\%$$

$$\mathrm{EE}\%:\frac{\text{mass of antigen encapsulated by microparticles}}{\text{initial mass of antigen in the loading solution}}\times 100\%$$

### 4.2 Fabrication of microneedle patches

MNP fabrication methods have been described previously and their adaptation to this study is summarized here^45^ The patches were fabricated by casting onto polydimethtylsioxane (PDMS) molds to produce MNPs containing a 10 × 10 array of pyramidal microneedles (300 × 300 × 600 μm^3^) with tip‐to‐tip spacing at 640 μm.

To make standard MNPs (lacking a pedestal), antigen‐loaded microparticles were first washed 3× with MOPS, then resuspended in cold ddH_2_O at an approximate concentration of 40 mg/ml and kept on ice. About 25 μl of the microparticle suspension was pipetted onto the surface of the PDMS mold, and the mold was pulled under vacuum for 10 min at approximately 25 in.Hg. Excess suspension was then removed and returned to the stock for reuse. The mold was then centrifuged for 10 min at 3,220 rcf at 4 °C. Excess microparticles were removed from the surface of the mold via gentle tape‐stripping. Approximately 90 μl of a 40% PVA + 30% sucrose (wt/vol) solution was then applied over the molds, and pulled under vacuum for 30 min. The patches were then allowed to dry in a fume hood overnight before being demolded and trimmed of excess material around the edges to form a ∼1 cm^2^ square patch. The patches were then freeze‐dried for >48 hr. Patches were stored under desiccation at 4 °C until use.

Plastic pedestal masters were 3D printed with assistance from the University of Michigan 3D lab using a ProJet 3500 HD Max printer. The pedestal was prepared based on lithography methods previously described.^48^ It consisted of a 10 × 10 array that could be overlaid onto the MNP mold (equivalent center‐to‐center spacing of 640 μm), made of pyramidal trapezoids with a 300 μm wide square base, 800 μm tall, and a 130 μm wide square top. After fabrication the mold was cleaned of printing oil, then a PDMS mold was cast from the structure. This new mold allowed the pedestal part to be recreated using excipients with expected excellent biocompatibility (PVA/sucrose).

To create pedestal patches, the aforementioned patch process was carried out identically through the first centrifugation step. After tape‐stripping away excess surface microparticles, 25 μl of the PVA/sucrose mixture (as above) was vacuumed onto the mold for 10 min while the mold was covered to prevent evaporation and premature hardening of the patch. Surface PVA/sucrose was then removed using a razor under a stereomicroscope (Nikon Olympus). The PVA/sucrose pedestal part was then manually aligned with the microneedle cavities (still in the mold) such that the tip of each pedestal aligned with the tip of the microneedle molds. The pedestal was then gently pressed into the mold and was allowed to dry in‐place in a fume hood overnight. Patches were then demolded and freeze‐dried. Each patch used in this study was visualized on a stereomicroscope to ensure microneedle quality. Malformed patches were occasionally formed, but discarded.

### 4.3 In vitro release and stability

For in vitro evaluation of antigen‐loaded microparticles, microparticles were suspended in 1 ml PBST (PBS + 0.02% Tween 80). For MNPs, four patches were first dissolved in ddH_2_O and rinsed 5× to remove PVA/sucrose microneedle matrix material, then the remaining microparticles were resuspended in 0.25 ml PBST. Release studies were carried out at 37 °C while protected from light and shaken at 240 rpm. At each timepoint (1, 3, 7 days and weekly thereafter), samples were spun‐down and the full release media was removed for antigen analysis via HPLC/UPLC‐SEC and/or ELISA.

### 4.4 Size exclusion Chromatogaphy of antigens

Unless otherwise stated, antigen concentration was determined by SEC using either high or ultra performance liquid chromatography (HPLC/UPLC). In either case, the mobile phase consisted of PBS, pH 7.4, flowed at 1 ml/min (HPLC) or 0.4 ml/min (UPLC). Injection volumes were 50 or 10 μl for HPLC and UPLC, respectively. All samples were filtered through 0.45 μm filters prior to injection. A TSKgel G3000SWxl column was used for HPLC and an Acquity BEH SEC (4.6 × 150 mm) column was used for UPLC. UV detection was done at 215 nm. All samples were carried out in triplicate or greater, and only monomeric protein content was considered.

### 4.5 Total nitrogen analysis

Total protein content was extrapolated from total nitrogen content using a modified automated Dumas technique.^62^ Microparticle pellets were washed 3× with ddH_2_O, then freeze‐dried. About 1–4 mg of microparticles were massed into tin pans, which were crimped to remove excess air. Samples were run on a Leco TrueSpec Micro CHN. The instrument was first blanked without samples to establish atmospheric baselines. Carbon, hydrogen, and nitrogen standards were then set in the anticipated range of nitrogen mass using USP‐grade EDTA. Lyophilized antigen standards were run to verify the percent nitrogen in the protein and set a Protein Factor. Microparticle samples were then dropped into the combustion chamber at 1,050 °C, which converts all nitrogen to nitrogen gas, which is then quantified by a thermal conductivity cell. Protein content was determined by multiplying the nitrogen mass by the protein factor after first subtracting the nitrogen mass from negative controls (unloaded microparticles). Percent protein could then be determined by dividing protein mass by total sample mass.

### 4.6 Confocal microscopy

To visualize the distribution of antigen inside the microparticles after encapsulation, microparticles were loaded using an Ovalbumin‐Alexa Fluor 647 conjugate (fOVA) similar to as described above. After washing, the microparticles were resuspended in ddH_2_O and placed on a glass slide with a coverslip and cross‐sectional Z‐stacked images were taken on a Nikon A‐1 spectral confocal laser scanning microscope (CLSM) operating with a Cy5 filter and NIS Elements viewing and analysis software.

To evaluate the particulate release fraction, fOVA‐loaded microparticles were resuspended in PBST at 37 °C. At predetermined time points, a sample of the suspension was removed and washed with ddH_2_O before similarly imaging as above via CLSM. Images were compared against Alhydrogel that had similarly been loaded with fOVA and washed of unbound antigen.

### 4.7 Microneedle insertion

For ex vivo evaluation of mechanical integrity, excised porcine ear tissue was used. The shaved inner skin with cartilage attached was separated from the outer skin and subcutaneous fat, and pinned taut. MNPs were gently placed tip‐down onto the skin, and pressed in firmly with the thumb for 10 s. The patch was then removed and Gentian Violet (Ricca Chemical Co.) was applied to the application site for 1 min before being wiped away with an alcohol pad. The application site was then cut away and imaged on a stereomicroscope (*n* = 5 for each patch type).

To evaluate depth of penetration/microparticle deposition, microparticles loaded with OVA‐AlexaFluor 488 conjugate were fabricated into MNPs and the experiment was performed similar to above, except patches were held on the tissue for 5 min with pressure, then placed in a 37 °C chamber at 98% humidity for 15 additional minutes to allow the microneedless to dissolve. The backing of the patches was gently removed and the application site tissue was cut out and embedded in OCT compound, which was subsequently dipped in isopentane chilled by surrounding LN_2_. The samples were then cut into 50 μm sections using a Leicia 3050S cryostat onto Superfrost+ microscope slides. Slides were thawed and immediately imaged on an Olympus fluorescent stereomicroscope.

To determine the mass of microparticles delivered upon application of MNPs, male nude BALB/c mice were cleared of any light hair using depilatory cream (Nair) 1 day in advance of patch application. Mice were anesthetized and placed on a heated pad to maintain body temperature. A fold of skin from the dorsal flank was pulled from the body and held taut on a cutting board. A MNP was gently pressed into the skin for 5 min. Pressure was then removed and the patch was kept on the skin for an additional 15 min. The remaining portion of the patch was then removed and placed in a microcentrifuge tube. Four patches were used per sample (*n* = 3 samples). The patches were then dissolved in ddH_2_O and washed 5×, then dried in a vacuum oven at 40 °C overnight. To account for residual animal tissue that was picked up by the patches, the mass of microparticles remaining in the patches after application was determined by gel permeation chromatography (GPC). Briefly, the residual microparticles were dissolved in tetrahydrofuran, filtered, and ran on a GPC column against standard masses of dissolved microparticles.

### 4.8 In vivo microparticle tracking

The treatment of all experimental animals in these procedures were in accordance with University committee on use and care of animals (University of Michigan UCUCA), and all NIH guidelines for the care and use of laboratory animals. Pedestal MNPs were made loaded with fOVA and applied to male albino C57BL/6J mice as described above. Two patches were applied per mouse, to the left anterior and right posterior dorsal flank. At predetermined time‐points, the whole animal was anesthetized and imaged using a PerkinElmer IVIS Spectrum imaging system. Fluorescence data was processed using a region‐of‐interest analysis with background subtraction using Living Image 4.5 software. Other study groups included mice given an i.d. injection to the same locations of an equivalent delivered dose of fOVA‐loaded microparticles or soluble fOVA. Mice were kept on an alfalfa‐free diet to reduce autofluorescence. Depilatory cream was not reapplied during the study, but hair was kept trimmed using electric razors (*n* = 4 mice/group, two applications per mouse).

### 4.9 Skin resealing

TEWL was measured using a Delfin Technologies VapoMeter with DelfWin 4 capture software. Study groups consisted of application of: (a) PLA master patches (no pedestal), (b) ASE microparticle‐loaded pedestal patches, (c) pedestal patches loaded with smaller nano‐sized PLGA particles,^59^ and (d) vehicle‐only patches (pedestal MNPs made of only PVA/sucrose, no microparticles). Three measurements were taken per application site, per animal, at each timepoint, and the TEWL chamber was allowed to re‐equilibrate to environmental conditions before each measurement. To measure TEWL, the VapoMeter was gently pressed against the application site without manual tension applied to the skin. Data are presented as percent increase over an application control using ANOVA with Fisher's LSD. The application control consisted of a flat PVA/sucrose mock patch that did not contain any microneedles, but was applied similarly to other groups.

### 4.10 Immunizations

C57Bl/6 (for OVA groups) or BALB/c (for rHBsAg groups) mice, 5–6 weeks old, five mice/group, were purchased from Jackson Laboratories. The choice of mouse strain was reliant on reagents available for the different antigens. One day prior to priming and booster immunization the application site for MN patches or i.d. administered groups was shaved and depilatory cream was applied, or just shaved for i.m. administered groups. On day zero mice were immunized with either: (a) two pedestal MNPs, or equivalent delivered antigen dose from, (b) i.d. microparticles, (c) i.m. microparticles, (d) Alhydrogel‐adsorbed antigen, or (e) soluble antigen. A sham group received patches containing microparticles that did not contain antigen. Booster doses were given 21 days after the priming dose.

To evaluate antibody titers, blood was drawn on days 20 and 42 via submandibular bleed. Serum was separated using Microvette 500 Zgel serum collection tubes centrifuged for 5 min at 10,000 rcf. Serum was stored at −80 °C until analysis. Serum samples were analyzed by the University of Michigan Cancer Center Immunology Core for IgG, IgG1, and IgG2c via ELISA. Due to reagent availability, antigen‐specific IgG1 isotype was used as a standard for all IgGs to determine relative concentration. Data were compared using one‐way ANOVA with Tukey's post‐test via GraphPad Prism software.

To evaluate the nature of the cytokine response produced after restimulation of splenic lymphocytes, all mice were euthanized on day 42 and spleens were collected under sterile conditions. Splenocytes were collected by grinding each spleen through a 70 μm nylon strainer. Red blood cells were lysed with ACK lysing buffer and the cells were washed 3× with sterile PBS before being resuspended in RPMI 1640 media supplemented with glutamine, 10% FBS (10%), 1 U/ml penicillin + 1 μg/ml streptomycin, 55 μM 2‐mercaptoethanol, MEM nonessential amino acids (1%), 1 mM sodium pyruvate, and 10 mM HEPES. Cells were then plated at 5 × 10^5^ cells/well in a 96‐well plate and stimulated with media (negative control) or 25 μg/ml whole antigen (OVA or rHBsAg). Positive controls were pooled from each spleen within a group and stimulated with 2 μl/ml PMA/ionomycin (cell stimulation cocktail). Cells were incubated for 96 hr at 37 °C with 5% CO_2_ before collecting the supernatant and storing at −80 °C. Concentrations of IL2, IL6, IL10, and TNFα were analyzed via ELISA through the University of Michigan Cancer Center Immunology Core. Stimulated cell supernatants were compared against negative controls using Student's *t*‐test.

## Supporting information

Appendix S1: Supporting Information for Self‐healing Encapsulation and Controlled Release of Vaccine Antigens from PLGA Microparticles Delivered by Microneedle Patches

## References

[1] Glenn GM , Kenney RT . Mass vaccination: solutions in the skin. Curr Top Microbiol. 2006;304:247‐268. 10.1007/3-540-36583-4_14 16989274

[2] Belshe RB , Newman FK , Wilkins K , et al. Comparative immunogenicity of trivalent influenza vaccine administered by intradermal or intramuscular route in healthy adults. Vaccine. 2007;25(37–38):6755‐6763. 17692438 10.1016/j.vaccine.2007.06.066 PMC2148502

[3] Koutsonanos DG , Compans RW , Skountzou I . Targeting the skin for microneedle delivery of influenza vaccine. Adv Exp Med Biol. 2013;785:121‐132. 23456844 10.1007/978-1-4614-6217-0_13 PMC6525635

[4] Marshall S , Sahm LJ , Moore AC . The success of microneedle‐mediated vaccine delivery into skin. Hum Vaccin Immunother. 2016;12(11):2975‐2983. 27050528 10.1080/21645515.2016.1171440 PMC5137519

[5] Cleland JL . Single‐administration vaccines: controlled‐release technology to mimic repeated immunizations. Trends Biotechnol. 1999;17(1):25‐29. 10098275 10.1016/s0167-7799(98)01272-4

[6] Kim YC , Park JH , Prausnitz MR . Microneedles for drug and vaccine delivery. Adv Drug Deliv Rev. 2012;64(14):1547‐1568. 22575858 10.1016/j.addr.2012.04.005 PMC3419303

[7] Prausnitz MR , Langer R . Transdermal drug delivery. Nat Biotechnol. 2008;26(11):1261‐1268. 18997767 10.1038/nbt.1504 PMC2700785

[8] Norman JJ , Arya JM , McClain MA , Frew PM , Meltzer MI , Prausnitz MR . Microneedle patches: usability and acceptability for self‐vaccination against influenza. Vaccine. 2014;32:1856‐1862. 24530146 10.1016/j.vaccine.2014.01.076 PMC3979961

[9] Indermun S , Luttge R , Choonara YE , et al. Current advances in the fabrication of microneedles for transdermal delivery. J Control Release. 2014;185:130‐138. 24806483 10.1016/j.jconrel.2014.04.052

[10] Lofthouse S . Immunological aspects of controlled antigen delivery. Adv Drug Deliv Rev. 2002;54(6):863‐870. 12363435 10.1016/s0169-409x(02)00073-x

[11] O'Hagan DT , Singh M , Gupta RK . Poly(lactide‐co‐glycolide) microparticles for the development of single‐dose controlled‐release vaccines. Adv Drug Deliv Rev. 1998;32(3):225‐246. 10837646

[12] Kohn J , Niemi SM , Albert EC , Murphy JC , Langer R , Fox JG . Single‐step immunization using a controlled release, biodegradable polymer with sustained adjuvant activity. J Immunol Methods. 1986;95(1):31‐38. 3782824 10.1016/0022-1759(86)90314-5

[13] Sanchez A , Gupta RK , Alonso MJ , Siber GR , Langer R . Pulsed controlled‐release system for potential use in vaccine delivery. J Pharm Sci. 1996;85(6):547‐552. 8773947 10.1021/js960069y

[14] Kemp JM , Kajihara M , Nagahara S , Sano A , Brandon M , Lofthouse S . Continuous antigen delivery from controlled release implants induces significant and anamnestic immune responses. Vaccine. 2002;20(7–8):1089‐1098. 11803069 10.1016/s0264-410x(01)00444-3

[15] Johansen P , Storni T , Rettig L , et al. Antigen kinetics determines immune reactivity. Proc Natl Acad Sci USA. 2008;105(13):5189‐5194. 18362362 10.1073/pnas.0706296105 PMC2278203

[16] Wischke C , Schwendeman SP . Degradable polymeric carriers for parenteral controlled drug delivery In: Siepmann J, Siegel RA, Rathbone MJ, eds. Fundamentals and Applications of Controlled Release Drug Delivery. Boston, MA: Springer US; 2012:171‐228.

[17] Schwendeman SP , Costanitino HR , Gupta RK , Langer R . Peptide, protein, and vaccine delivery from implantable polymeric systems In: Controlled Drug Delivery, Challenges and Strategies. Washington, DC: American Chemical Society; 1997:229‐267.

[18] Hoffman AS . The origins and evolution of "controlled" drug delivery systems. J Control Release. 2008;132(3):153‐163. 18817820 10.1016/j.jconrel.2008.08.012

[19] Schwendeman SP , Costantino HR , Gupta RK , Peptide LR . Protein, and vaccine delivery from implantable polymeric systems progress and challenges In: Park K, ed. Controlled Drug delivery: Challenges and Strategies. Washington DC: American Chemical Society; 1997:229‐267.

[20] Schwendeman SP . Recent advances in the stabilization of proteins encapsulated in injectable PLGA delivery systems. Crit Rev Ther Drug Carrier Syst. 2002;19(1):73‐98. 12046892 10.1615/critrevtherdrugcarriersyst.v19.i1.20

[21] Maa YF , Hsu CC . Protein denaturation by combined effect of shear and air‐liquid interface. Biotechnol Bioeng. 1997;54(6):503‐512. 18636406 10.1002/(SICI)1097-0290(19970620)54:6<503::AID-BIT1>3.0.CO;2-N

[22] Sah H . Protein behavior at the water/methylene chloride interface. J Pharm Sci. 1999;88(12):1320‐1325. 10585229 10.1021/js9900654

[23] Alexander P , Hamilton LD , Stacey KA . Irradiation of proteins in the solid state. I. Aggregation and disorganization of secondary structure in bovine serum albumin. Radiat Res. 1960;12:510‐525. 13792598

[24] Desai KG , Schwendeman SP . Active self‐healing encapsulation of vaccine antigens in PLGA microspheres. J Control Release. 2013;165(1):62‐74. 23103983 10.1016/j.jconrel.2012.10.012 PMC3888863

[25] Shah RB , Schwendeman SP . A biomimetic approach to active self‐microencapsulation of proteins in PLGA. J Control Release. 2014;196:60‐70. 25219750 10.1016/j.jconrel.2014.08.029 PMC4268178

[26] Reinhold SE , Desai KG , Zhang L , Olsen KF , Schwendeman SP . Self‐healing microencapsulation of biomacromolecules without organic solvents. Angew Chem Int Ed Engl. 2012;51(43):10800‐10803. 23011773 10.1002/anie.201206387 PMC3817563

[27] Huang J , Mazzara JM , Schwendeman SP , Thouless MD . Self‐healing of pores in PLGAs. J Control Release. 2015;206:20‐29. 25701611 10.1016/j.jconrel.2015.02.025

[28] Donnelly RF , Raj Singh TR , Woolfson AD . Microneedle‐based drug delivery systems: microfabrication, drug delivery, and safety. Drug Deliv. 2010;17(4):187‐207. 20297904 10.3109/10717541003667798 PMC2906704

[29] Park JH , Allen MG , Prausnitz MR . Polymer microneedles for controlled‐release drug delivery. Pharm Res. 2006;23(5):1008‐1019. 16715391 10.1007/s11095-006-0028-9

[30] Gill HS , Denson DD , Burris BA , Prausnitz MR . Effect of microneedle design on pain in human volunteers. Clin J Pain. 2008;24(7):585‐594. 18716497 10.1097/AJP.0b013e31816778f9 PMC2917250

[31] Haq MI , Smith E , John DN , et al. Clinical administration of microneedles: skin puncture, pain and sensation. Biomed Microdevices. 2009;11(1):35‐47. 18663579 10.1007/s10544-008-9208-1

[32] Arya J , Prausnitz MR . Microneedle patches for vaccination in developing countries. J Control Release. 2016;240:135‐141. 26603347 10.1016/j.jconrel.2015.11.019 PMC4871790

[33] Quan FS , Kim YC , Vunnava A , et al. Intradermal vaccination with influenza virus‐like particles by using microneedles induces protection superior to that with intramuscular immunization. J Virol. 2010;84(15):7760‐7769. 20484519 10.1128/JVI.01849-09 PMC2897640

[34] Kim YC , Quan FS , Compans RW , Kang SM , Prausnitz MR . Formulation and coating of microneedles with inactivated influenza virus to improve vaccine stability and immunogenicity. J Control Release. 2010;142(2):187‐195. 19840825 10.1016/j.jconrel.2009.10.013 PMC2823933

[35] Mazzara JM , Balagna MA , Thouless MD , Schwendeman SP . Healing kinetics of microneedle‐formed pores in PLGA films. J Control Release. 2013;171(2):172‐177. 23831588 10.1016/j.jconrel.2013.06.035

[36] Blasi P , D'Souza SS , Selmin F , DeLuca PP . Plasticizing effect of water on poly(lactide‐co‐glycolide). J Control Release. 2005;108(1):1‐9. 16098624 10.1016/j.jconrel.2005.07.009

[37] Champion JA , Walker A , Mitragotri S . Role of particle size in phagocytosis of polymeric microspheres. Pharm Res. 2008;25(8):1815‐1821. 18373181 10.1007/s11095-008-9562-y PMC2793372

[38] Baylor NW , Egan W , Richman P . Aluminum salts in vaccines—US perspective. Vaccine. 2002;20(suppl 3):S18‐S23. 12184360 10.1016/s0264-410x(02)00166-4

[39] Marrack P , McKee AS , Munks MW . Towards an understanding of the adjuvant action of aluminium. Nat Rev Immunol. 2009;9(4):287‐293. 19247370 10.1038/nri2510 PMC3147301

[40] Shirodkar S , Hutchinson RL , Perry DL , White JL , Hem SL . Aluminum compounds used as adjuvants in vaccines. Pharm Res. 1990;7(12):1282‐1288. 2095567 10.1023/a:1015994006859

[41] Jiang G , Joshi SB , Peek LJ , et al. Anthrax vaccine powder formulations for nasal mucosal delivery. J Pharm Sci. 2006;95(1):80‐96. 16315230 10.1002/jps.20484

[42] Demuth PC , Garcia‐Beltran WF , Ai‐Ling ML , Hammond PT , Irvine DJ . Composite dissolving microneedles for coordinated control of antigen and adjuvant delivery kinetics in transcutaneous vaccination. Adv Funct Mater. 2013;23(2):161‐172. 23503923 10.1002/adfm.201201512 PMC3595545

[43] Zaric M , Lyubomska O , Poux C , et al. Dissolving microneedle delivery of nanoparticle‐encapsulated antigen elicits efficient cross‐priming and Th1 immune responses by murine Langerhans cells. J Invest Dermatol. 2015;135(2):425‐434. 25243789 10.1038/jid.2014.415

[44] Larraneta E , McCrudden MT , Courtenay AJ , Donnelly RF . Microneedles: a new frontier in nanomedicine delivery. Pharm Res. 2016;33(5):1055‐1073. 26908048 10.1007/s11095-016-1885-5 PMC4820498

[45] Chu LY , Choi SO , Prausnitz MR . Fabrication of dissolving polymer microneedles for controlled drug encapsulation and delivery: bubble and pedestal microneedle designs. J Pharm Sci. 2010;99(10):4228‐4238. 20737630 10.1002/jps.22140

[46] Naito S , Ito Y , Kiyohara T , Kataoka M , Ochiai M , Takada K . Antigen‐loaded dissolving microneedle array as a novel tool for percutaneous vaccination. Vaccine. 2012;30(6):1191‐1197. 22172508 10.1016/j.vaccine.2011.11.111

[47] Chu LY , Prausnitz MR . Separable arrowhead microneedles. J Control Release. 2011;149(3):242‐249. 21047538 10.1016/j.jconrel.2010.10.033 PMC3040254

[48] Chen MC , Huang SF , Lai KY , Ling MH . Fully embeddable chitosan microneedles as a sustained release depot for intradermal vaccination. Biomaterials. 2013;34(12):3077‐3086. 23369214 10.1016/j.biomaterials.2012.12.041

[49] Morefield GL , Jiang D , Romero‐Mendez IZ , Geahlen RL , Hogenesch H , Hem SL . Effect of phosphorylation of ovalbumin on adsorption by aluminum‐containing adjuvants and elution upon exposure to interstitial fluid. Vaccine. 2005;23(12):1502‐1506. 15670886 10.1016/j.vaccine.2004.08.048

[50] Hem SL , Hogen EH . Aluminum‐containing adjuvants: properties, formulation, and use In: Singh M, ed. Vaccine Adjuvants and Delivery Systems. Hoboken, NJ: John Wiley & Sons; 2007:81‐114.

[51] Hansen B , Belfast M , Soung G , et al. Effect of the strength of adsorption of hepatitis B surface antigen to aluminum hydroxide adjuvant on the immune response. Vaccine. 2009;27(6):888‐892. 19071182 10.1016/j.vaccine.2008.11.078

[52] Iyer S , Robinett RS , HogenEsch H , Hem SL . Mechanism of adsorption of hepatitis B surface antigen by aluminum hydroxide adjuvant. Vaccine. 2004;22(11–12):1475‐1479. 15063571 10.1016/j.vaccine.2003.10.023

[53] Lee JW , Choi SO , Felner EI , Prausnitz MR . Dissolving microneedle patch for transdermal delivery of human growth hormone. Small. 2011;7(4):531‐539. 21360810 10.1002/smll.201001091 PMC4143249

[54] Kalluri H , Kolli CS , Banga AK . Characterization of microchannels created by metal microneedles: formation and closure. AAPS J. 2011;13(3):473‐481. 21732220 10.1208/s12248-011-9288-3 PMC3160154

[55] Donnelly RF , Singh TR , Tunney MM , et al. Microneedle arrays allow lower microbial penetration than hypodermic needles in vitro. Pharm Res. 2009;26(11):2513‐2522. 19756972 10.1007/s11095-009-9967-2 PMC2900181

[56] Gupta J , Gill HS , Andrews SN , Prausnitz MR . Kinetics of skin resealing after insertion of microneedles in human subjects. J Control Release. 2011;154(2):148‐155. 21640148 10.1016/j.jconrel.2011.05.021 PMC3164267

[57] Rovee DT , Maibach HI . The epidermis in wound healing. Boca Raton, FL: CRC Press; 2004.

[58] Park JH , Allen MG , Prausnitz MR . Biodegradable polymer microneedles: fabrication, mechanics and transdermal drug delivery. J Control Release. 2005;104(1):51‐66. 15866334 10.1016/j.jconrel.2005.02.002

[59] Bailey B . Development and Characterization of Novel Self‐Encapsulating Poly(lactic‐co‐glycolic acid) Microspheres for Vaccine Delivery. Ph.D. thesis, University of Michigan, Ann Arbor, MI; 2016.

[60] Clausen OP , Schjolberg AR . Turnover and maturation kinetics in regenerating mouse epidermis. Apmis. 1988;96:111‐119. 3179031

[61] Storni T , Kundig TM , Senti G , Johansen P . Immunity in response to particulate antigen‐delivery systems. Adv Drug Deliv Rev. 2005;57(3):333‐355. 15560945 10.1016/j.addr.2004.09.008

[62] Etheridge RD , Pesti GM , Foster EH . A comparison of nitrogen values obtained utilizing the Kjeldahl nitrogen and Dumas combustion methodologies (Leco CNS 2000) on samples typical of an animal nutrition analytical laboratory. Anim Feed Sci Tech. 1998;73(1–2):21‐28.

