# Supplementary material for: Self‐healing encapsulation and controlled release of vaccine antigens from PLGA microparticles delivered by microneedle patches
Source: Bioeng Transl Med. 2018 Oct 30;4(1):116–28. doi: 10.1002/btm2.10103 (PMC6336668; doi:10.1002/btm2.10103)
Supplement: Supplementary file 1 — Appendix S1: Supporting Information for Self‐healing Encapsulation and Controlled Release of Vaccine Antigens from PLGA Microparticles Delivered by Microneedle Patches [file BTM2-4-116-s001.pdf]

Supporting Information

S.1 Splenic Cytokine Release: To evaluate the nature of the cytokine response produced after re-stimulation of splenic lymphocytes, all mice were euthanized on day 42 and spleens were collected under sterile conditions. Splenocytes were collected by grinding each spleen through a 70 µm nylon strainer. Red blood cells were lysed with ACK lysing buffer and the cells were washed 3X with sterile PBS before being resuspended in RPMI 1640 media supplemented with glutamine, 10% FBS (10%), 1 U/mL penicillin + 1 µg/mL streptomycin, 55 µM 2-mercaptoethanol, MEM non-essential amino acids (1%), 1 mM Sodium Pyruvate, and 10 mM HEPES. Cells were then plated at 5 x 10^5 cells/well in a 96-well plate and stimulated with media (negative control) or 25 µg/mL whole antigen (OVA or rHBsAg). Positive controls were pooled from each spleen within a group and stimulated with 2 µL/mL PMA/ionomycin (cell stimulation cocktail). Cells were incubated for 96 hours at 37 °C with 5% CO_2 before collecting the supernatant and storing at -80 °C. Concentrations of IL2, IL6, IL10, and TNFα were analyzed via ELISA through the University of Michigan Cancer Center Immunology Core. Stimulated cell supernatants were compared against negative controls using Student's t-test.

S.2 Scanning Electron Microscopy: Surface morphology of PLGA microparticles was evaluated by mounting dry microparticles on double-sided carbon tape and imaging on a FEI Quanta 3D scanning electron microscope (SEM) operated in low vacuum mode at voltages of 5 or 10 kV. Images were captured on EDAX® software.

S.3 Modulated Differential Scanning Calorimetry: Glass-transition temperatures (T_g) were determined by modulated differential scanning calorimetry (mDSC). For dry T_g, approximately 5 mg of lyophilized microparticles were crimped in aluminum pans with a

non-hermetic lid. For hydrated T_g, samples were sent through the loading gamut described in the PLGA Microparticle Fabrication and Loading section to load OVA, then excess solution was removed to create a slurry, which was then transferred to aluminum pans crimped with a hermetic lid. Hydrated T_gs are expected to be mostly similar for the antigens evaluated here. Temperatures were ramped between 5°C and 80°C at 3°C/min, with a modulation amplitude of ±1.0°C/min and a period of 60s. All samples were subjected to a heat/cool/heat cycle and the 2nd T_g was reported as the midpoint of the exothermic event. The analysis was done using TA Trios software, and all experiments were performed in triplicate.

2nd mDSC Heat	T_g (°C) ± SEM
Neat Microparticles	46.5° ± 0.0
Loaded, Hydrated	32.6° ± 0.2

Figure S1: T_g of unloaded lyophilized microparticles, and of OVA loaded and hydrated microparticles. The T_g of neat microparticles is above typical ambient temperatures, while the hydrated T_g is below physiological temperature. n=3, ± SEM.

OVA Solution Volume, V (µL)	OVA Solution Concentration, C (µg/mL)	Loading %	Encapsulation Efficiency %
1000	250	0.88 ± .00	75.4 ± .00
1000	500	1.01 ± .01	47.9 ± .00
1000	1000	1.50 ± .01	30.8 ± .00
500	1000	1.64 ± .03	66.5 ± 1.4
300	1000	1.34 ± .02	90.7 ± 1.6

Figure S2: Optimization of self-healing encapsulation of OVA model antigen. *20 mg of ASE microspheres were incubated in a volume of OVA solution, V, at OVA concentration, C. This suspension was lightly mixed for 2 days at 4 °C, 1 day at room temperature and then 2 days at 42 °C. ± SEM.

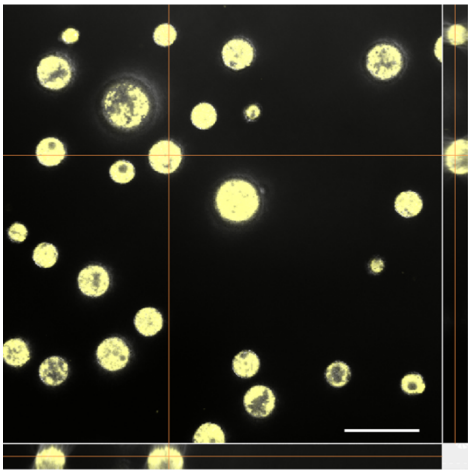

Figure S3: Active self-healing encapsulation results in thorough antigen distribution throughout the microparticles. CLSM depth profile of microparticles after loading with fOVA and healing at up to 42 °C. Scale = 100 µm.

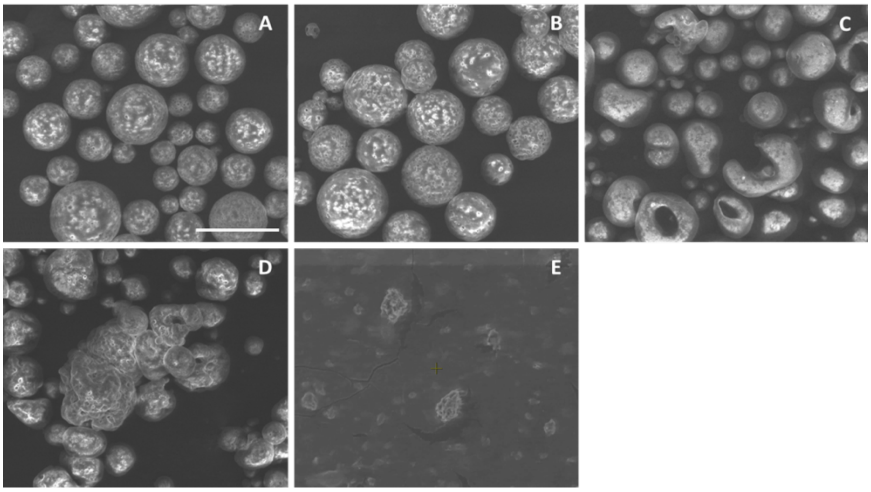

Figure S4: ASE microparticles slowly degrade during *in vitro* release at 37 °C, with significant degradation not apparent until 21 days. Microparticles after *A*) 7 d, *B*) 14 d, *C*) 21 d, *D*) 28 d, and *E*) 42 d in release. Scale = 50 µm.

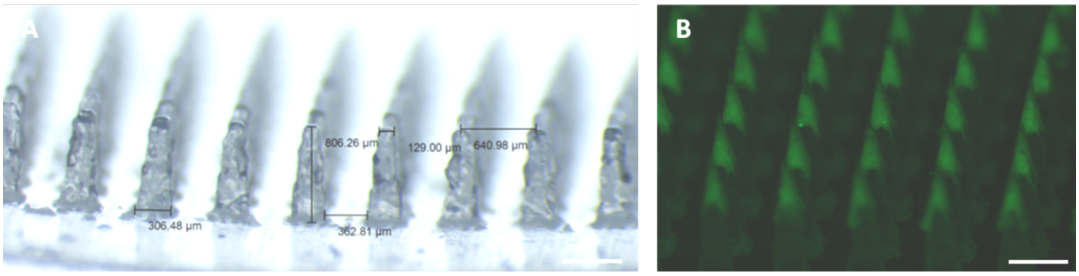

Figure S5: *A*) 3D printed pedestal master part. *B*) Fluorescent micrograph of pedestal patch loaded with fOVA-loaded microparticles. Scale = 500 µm.

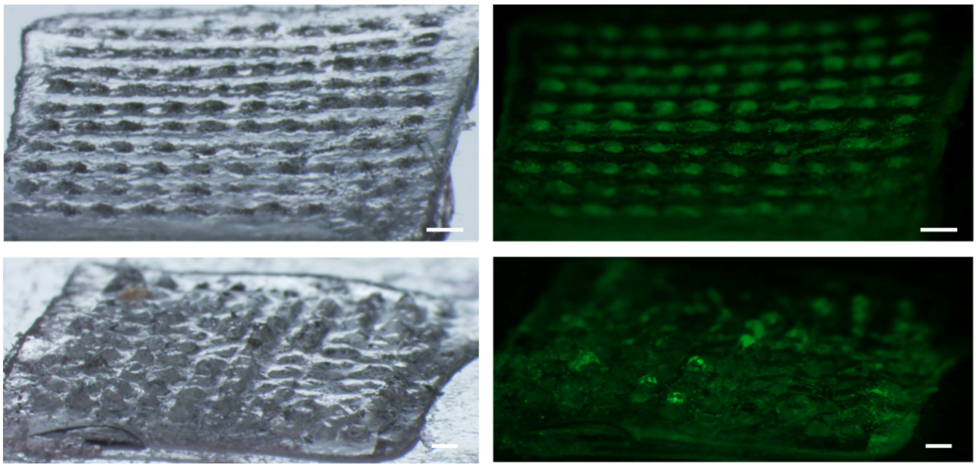

Figure S6: Stereomicrographs of (*Top*) standard, and (*Bottom*) pedestal patches after application to mice. Right images emphasize microparticles via fluorescent imaging.

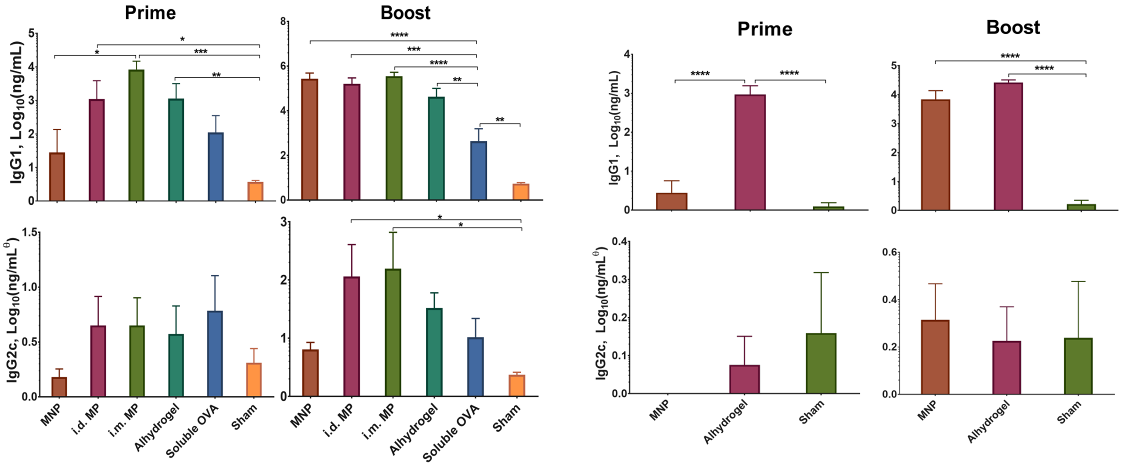

Figure S7: IgG1 and IgG2c levels for (*Left*) OVA and (*Right*) rHBsAg, 20 (Prime) and 42 (Boost) days after immunizations. Booster doses were given on day 21.
$^{\Theta}$ Concentrations were determined using an IgG1 standard, and may not be absolute for other IgG isotypes.
**** p <.0001, *** p < .001, ** p <.01, * p < .05. n=5, ± SEM.

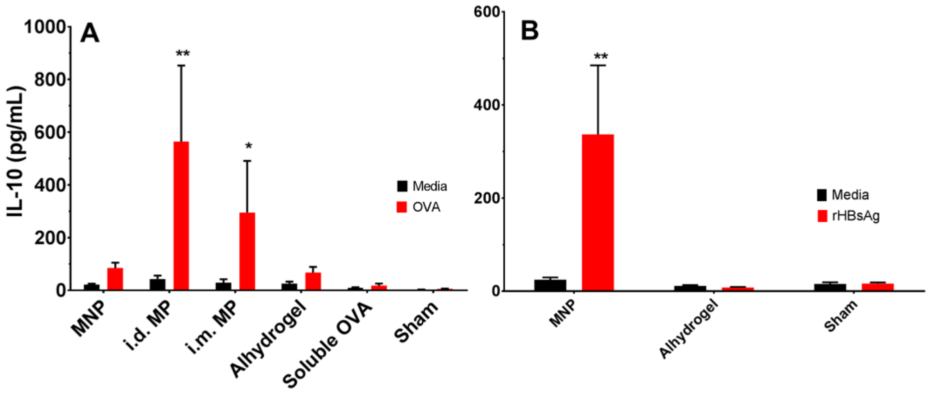

Figure S8: Splenocytes re-stimulated with OVA (*A*) or rHBsAg (*B*) produce considerable amounts of IL-10, indicative of a Th2-type immune response. ** p <.01, * p < .05. n=5, ± SEM.
